# How to Promote Urban Intelligent Transportation: A Fuzzy Cognitive Map Study

**DOI:** 10.3389/fnins.2022.919914

**Published:** 2022-07-06

**Authors:** Luwei Zhao, Qing’e Wang, Bon-Gang Hwang

**Affiliations:** ^1^School of Civil Engineering, Central South University, Changsha, China; ^2^Department of the Built Environment, College of Design and Engineering, National University of Singapore, Singapore, Singapore

**Keywords:** smart city, intelligent transportation, exploratory factor analysis, fuzzy cognitive map, critical variable

## Abstract

As an important part of smart city, intelligent transportation is an critical breakthrough to solve urban traffic congestion, build an integrated transportation system, realize the intelligence of traffic infrastructure and promote sustainable development of traffic. In order to investigate the construction of intelligent transportation in cities, 20 initial affecting variables were determined in this study based on literature analysis. A questionnaire collected from professionals in intelligent transportation was conducted, and a total of 188 valid responses were received. Then the potential grouping was revealed through exploratory factor analysis. Finally, a causal model containing seven concepts was established using the practical experience and knowledge of the experts. A root cause analysis method based on fuzzy cognitive map (FCM) was also proposed to simulate intelligent transportation construction (ITC). The results indicate:(1) The 20 variables can be divided into six dimensions: policy support (PS), traffic sector control (TSC), technical support (TS), communication foundation (CF), residents’ recognition (RR), and talent quality (TQ); and (2) In the FCM model, all six concept nodes (PS, TSC, TS, CF, RR, and TQ) have a significant positive correlation with the target concept node ITC. The rank of the six dimensions according to correlation strength is TS, CF, PS, TSC, RR, and TQ. The findings of this paper can help academics and practitioners understand the deep-seated determinants of urban intelligent transportation construction more comprehensively, and provide valuable suggestions for policy makers. And thus, the efficiency of intelligent transportation construction can be improved.

## Introduction

With the acceleration of urbanization, the pilot constructions of smart cities in China are actively under way. Smart cities collect and analyze data in real time using interconnection technology and make prediction and adaptive decisions to improve functional efficiency (Albino et al., 2015; Nikitas et al., 2020; Chakraborty et al., 2021). Since the faster population growth rate, the urban traffic congestion problem is still an inevitable problem in the current urbanization process, and the growth rate of per capita road area is still relatively slow, although the urban road construction developed rapidly presently. Generally speaking, traffic congestion may induce economic losses and other inevitable problems. Therefore, smart transportation construction is becoming an important part of smart city construction. Intelligent transportation system (ITS) is also becoming a booming field, making urban traffic safer and more efficient (Zhu et al., 2018).

The development of ITS initiated from the early 1970s, its purpose is to provide excellent services for the drivers and passengers in the transportation system. As the development direction of future transportation system, ITS combines advanced technologies, including electronic sensor technology, data transmission technology and intelligent control technology (Qi, 2008; An et al., 2011; Mayilvaganan and Sabitha, 2013; Shi and Abdel-Aty, 2015).

There are many definitions of ITS. Some scholars define it as a system using Internet, mobile communications, big data, cloud computing, computing intelligence and analysis technology to improve road safety and manage traffic (Winkowska et al., 2019). It is also defined as an system that integrates communication, control, vehicle sensing and electronic technologies to address traffic-related challenges (Ajayi et al., 2021). Some scholars defined it as a system composed of three main parts: traffic infrastructure, traffic management system, information and communication technology (Singh and Gupta, 2015). The transportation infrastructure includes road network, cars, buses, traffic lights and so on. Traffic management system includes regulatory agencies, traffic rules and so on. Information and communication technologies include the Internet, cloud / fog / edge computing, cellular networks (3G/4G/5G) and global positioning systems (GPS) (Wahab et al., 2020). Without exception, all the definitions mentioned above embed the information and communication technology into the transport system. ITS can also realize intelligent traffic management through intelligent parking (Bagula et al., 2015), intelligent traffic lights, vehicle monitoring and tracking, accident detection, license plate recognition, path planning, real-time infrastructure management and so on (Adeniran, 2017; Winkowska et al., 2019; Ajayi et al., 2021).

Based on the systematic literature review and practical analysis, Cledou et al. (2018) reviewed 42 publications about intelligent transportation service provided by nine intelligent cities around the world, and proposed a classification method for planning and designing intelligent transportation service. The proposals provide tools for policy makers and scenarios for the use of policies. The highly interconnected transportation system enables smart cities to detect emergency and make a response rapidly, improve efficiency and reduce risk greatly (Ganin et al., 2019). Simultaneously, interconnectedness itself presents a new vulnerability (Lombardi et al., 2012; Westraadt and Calitz, 2020), namely systems communicating with other systems and controlled remotely are easily hijacked (Petit and Shladover, 2014; Amoozadeh et al., 2015; Li et al., 2016). The network efficiency and resilience of random and targeted interruptions in ITS system have been studied in 10 urban areas, the results showed that under different threaten scenarios, the crossroads or roads controlled by the intelligent transportation system would be disturbed. Therefore, the flexible construction of transportation infrastructure should be investigated (Yang and Pun-Cheng, 2018; Ganin et al., 2019; Boukerche and Wang, 2020; Guevara and Auat Cheein, 2020; Haydari and Yilmaz, 2020).

In summary, the pioneer researches were mainly dedicated in the definition, development status and top-level design, construction mode of ITS. However, the empirical researches on the development and dynamic mechanism of ITC are still less reported. Therefore, Therefore, this study aims to solve the following problems: How to define the affecting factors of the development of intelligent transportation construction? How to distinguish the key factors affecting the development of intelligent transportation construction and its mechanism through empirical analysis?

The fuzzy cognitive map (FCM) method can consider the system composition and internal feedback mechanism, simulate the ITS through modeling, evaluate the long-term evolution of urban intelligent transportation system construction and reveal the complex relationship among the influencing factors (Pluchinotta et al., 2019; Chen et al., 2020; Pereira et al., 2020; Bakhtavar et al., 2021). The novelty of this method is that it can simulate the dynamic variability of system behavior with time, and can analyze a variety of scenarios, including predictive analysis and diagnostic analysis (Khanzadi et al., 2018; Azar and Dolatabad, 2019; Ladeira et al., 2019; Vaz et al., 2021). At the same time, it has a scientific feedback mechanism, which can accurately simulate the whole complex dynamic changes of ITS. Some scholars used FCM to carry out engineering researches to provide new reference ideas. Based on the construction experience and knowledge of experts, Zhang et al. (2017) used FCM to establish a causal model containing nine concepts, and simulated the performance of tunnel boring machines and suggested strategies to improve the efficiency of tunnel construction. Luo et al., 2020a,b, 2021) analyzed the influencing factors of prefabricated construction cost and construction project governance, respectively, summarized the influence factors, and constructed a causality model composed of 9 nodes for evolution analysis by using fuzzy cognitive map method.

In this study, 20 variables affecting the development of intelligent transportation construction were identified based on literature analysis. Through questionnaire and exploratory factor analysis, the 20 variables were divided into 6 dimensions: policy support (PS), traffic sector control (TSC), technical support (TS), communication foundation (CF), residents’ recognition (RR), and talent quality (TQ). The causal weights of the key factors were obtained based on expert interviews. The causality model composed of seven nodes (PS, TSC, TS, CF, RR, TQ and ITC) was constructed and the evolution was analyzed using the fuzzy cognitive map method, and reasonable suggestions were proposed finally.

This study can provide important theoretical basis and decision support for the construction and development of urban intelligent transportation. This study is universal and can be replicated and extended to more cities. Intelligent transportation can alleviate the problems of traffic congestion, environmental pollution, traffic safety and energy consumption in large, medium and small cities in China to a certain extent. It helps to alleviate and improve urban traffic service level and service efficiency. The application of emerging technologies can improve the information management level in the transportation field. This can also promote the sustainable development of urban transport system.

The remainder of this article is organized as follows. Section “Literature Review” introduces literature review. Section “Methodology” introduces several research methods used. Section “Data Analysis” describes how variables are grouped. Section “Fuzzy Cognitive Map Model and Predictive Analysis” describes the modeling and analysis process, section “Discussion” is a discussion, and section “Conclusion” concludes the paper.

## Literature Review

The existing research on intelligent transportation construction mainly focuses on traffic basic resources, technical support, residents’ quality, government support and so on. Based on the literature analysis in these fields, the main factors affecting the development of intelligent transportation were summarized.

Urban traffic infrastructure construction is the foundation of intelligent transportation construction. Urban traffic basic resources include road utilization rate, traffic information collection equipment, traffic signal control system, number of vehicles and so on. The population and the number of vehicles in a city will affect the efficiency of road use, which also has a great impact on the development of intelligent transportation construction (Zhang et al., 2021). The traffic information collection equipment can collect real-time traffic information within the city, vehicle information at the entrance and exit of the city, parking space information, non-motor vehicles and pedestrian information and so on. It provides data support for the development of traffic information and promotes the continuous improvement of the traffic control system (Guo and Lv, 2022). In the urban traffic system, alleviating road congestion, dredging traffic flow, restraining traffic accidents and improving air quality mainly rely on the traffic signal control system, which is also the main part of the urban traffic system (Neelakandan et al., 2021; Ghanadbashi and Golpayegani, 2022).

Vehicle-to-vehicle communication technology is based on Wifi transmission, so that different vehicles can share information with each other in a timely manner, such as location and speed. Based on the background information processing, the data is timely integrated, transmitted, analyzed and fed back to help the driver reasonably allocate their time and plan the travel route. Therefore, communication technology has a certain impact on the development of intelligent transportation construction. Communication technology includes wireless network transmission speed (Zhang et al., 2018; Zhang and Lu, 2020), wireless network coverage rate (Huang et al., 2019), GPS vehicle positioning device (Balid et al., 2018), driving decision optimization device (Shao et al., 2019), traffic flow forecast and safety monitoring technology based on cloud platform (Li W. et al., 2021), etc.

Talents are a powerful guarantee for the construction and development of intelligent transportation. Making full use of talents, scientific research and other favorable conditions to speed up the pace of traffic management, technology research and patent research and development is conducive to promoting the construction and development of intelligent transportation (Han and Zhang, 2021; Lycourghiotis et al., 2021). Strengthening citizens’ understanding and recognition of smart traffic is helpful to promote and apply command traffic construction in cities (Guo and Pei, 2022). The construction of intelligent transportation is inseparable from the cooperation between various departments, the maintenance of enterprise reporting system and equipment, and the protection of people to equipment. These behaviors need strong sense of social responsibility as a support.

The government has the responsibility and obligation to guide the construction of intelligent transportation. The policy documents related to smart city construction and emerging industry development planning involve many aspects, including scientific research policy, traffic development planning, investment and financing policy, and traffic management policy. These documents can provide policy guidance for the construction and development of intelligent transportation. The stimulation of scientific research policy helps to integrate the resources of industries and scientific research institutions in the city, solve the problems of insufficient motivation for industrial development, and promote the transformation and upgrading of traditional industries such as automobile manufacturing (Sumalee and Ho, 2018). Traffic development planning guides the planning, construction, operation and management of traffic development (Yuan and Li, 2021). Smart transportation construction relies on wireless equipment, Internet, cloud computing and other related industries. This determines that its construction needs a lot of money, needs to bear high risk, and the return cycle is long. Therefore, it is necessary for the government to formulate appropriate investment and financing policies to promote relevant enterprises to obtain financing support (Singh et al., 2020). In this process, with the integration of the new generation of information technology and the transportation industry, many new formats and new models have emerged. In order to reasonably guide the development of new formats, it is urgent to adjust the management policy (Boukerche et al., 2020). Increasing policy publicity can promote more citizens and enterprises to join the construction of smart cities (Zhu et al., 2019).

By synthesizing the literature review, typical cases in the literature and intelligent transportation development report, the preliminary variable list related to intelligent transportation construction was selected and modified. These variables have been mentioned by many researchers in this field. Combined with the expert survey feedback mentioned in the section “Data Analysis” of this study, a total of 20 initial variables were identified, as shown in Table 1.

**TABLE 1:** The sources of the variables.

No.	Variables	Sources
V01	Road utilization ratio	Zhang et al., 2021; Wang et al., 2022
V02	Road Video Surveillance System	Pramanik et al., 2021; Pratama and Supangkat, 2021; Guo and Lv, 2022
V03	Wireless network transmission speed	Zhang et al., 2018; Jacob and Darney, 2021
V04	Number of vehicles	Zhang et al., 2021
V05	Traffic flow collection system	Chen et al., 2021; Vijayalakshmi et al., 2021; Guo and Lv, 2022
V06	Driving decision optimization device	Shao et al., 2019; Yin et al., 2021
V07	Coverage rate of wireless networks	Huang et al., 2019; Wang G. G. et al., 2021
V08	GPS vehicle positioning device	Balid et al., 2018; Bai et al., 2021
V09	Traffic flow prediction and monitoring technology	Li J. et al., 2021; Li J. et al., 2021
V10	Residents’ cognition of intelligent transportation	Lycourghiotis et al., 2021; Mohandu and Kubendiran, 2021
V11	Social responsibility consciousness	Guo and Pei, 2022
V12	Education level of talents	Du et al., 2021; Han and Zhang, 2021
V13	Residents’ transportation travel mode	Yang et al., 2018; Zhao et al., 2022
V14	Special talent training	Han and Zhang, 2021; Manias and Shami, 2021
V15	Population aging rate	Yang et al., 2018; Kanthavel et al., 2021; Wang J. et al., 2021
V16	Scientific research policy	Sumalee and Ho, 2018; Gohar and Nencioni, 2021
V17	Traffic development planning	Nama et al., 2021; Yuan and Li, 2021
V18	Investment and financing policy	Singh et al., 2020; Razmjoo et al., 2021
V19	Traffic management policy	Boukerche et al., 2020; Poon, 2021; Telang et al., 2021
V20	Magnitude of propaganda	Zhu et al., 2019; Dauvergne, 2021

## Methodology

### Questionnaire Survey

The survey strategies of this study are as follow: (1) Extensive initial variables were selected through a comprehensive literature review. (2) Intelligent transportation development reports and institutional documents of some smart cities were collected and analyzed to supplement initial variables. (3) Before the comprehensive survey, five professionals conducted a pilot study by interviews to test and improve the initial variable list. All professionals have participated in the construction of intelligent transportation in many cities and have more than 10 years of research experience in the field of transportation. The pilot study has two main purposes: to test whether 20 variables are suitable for intelligent transportation construction and test whether the description or explanation of each variable is appropriate. The questionnaire was modified according to their feedback. (4) Conduct comprehensive investigations. The respondents included transportation department managers, workers involved in construction, subway drivers, taxi drivers, bus drivers, passengers, graduate students and teachers majoring in transportation. Some construction workers and drivers have college degrees or less, but they also have rich traffic experience and can make effective suggestions. The questionnaire is divided into two parts. The first part is to collect the basic information of participants in intelligent transportation, including age, working years, positions, education level, enterprise type and scale. The second part is used to measure the dimension division of influencing variables of intelligent transportation construction. A Likert 5-point scale is used to obtain respondents’ views on the importance of each variable, 1 being the least important and 5 the most important. A brief description of some unusual variables is also attached to the questionnaire to ensure that all respondents use the same definition of each variable. Participants were encouraged to add additional variables not mentioned in the list based on their experience. In this step, WeChat was used to distribute electronic questionnaires and recycle them. (5) SPSS 22.0 was used for exploratory factor analysis to identify important variables and reveal potential components.

### Fuzzy Cognitive Map

Fuzzy cognitive map is a fuzzy graph structure that allows causal propagation in the system, and its particularity is to allow forward and backward links. The unique reasoning technology of FCM enables it to simulate, analyze and evaluate the performance of complex systems under the consideration of system uncertainty, dynamics, interaction and interdependence. FCM is a dynamic system containing directed topology, which is composed of nodes, arcs, loops and feedback. The concept node is used to describe the concept of system behavior. The concept nodes are connected by symbols and weighted arcs to represent the causal relationship between concepts, including three steps of model design, establishment and application. Details are as follow: (1) Model design. Based on literature review, expert discussion, questionnaire survey and analysis, the key influencing factors of smart traffic construction were identified as nodes in dynamic fuzzy cognitive map. The 9-level fuzzy semantic scoring standard was established, and the causal relationship between the indicators is preliminarily identified. (2) Model establishment. Five experts were invited to assign the causal relationship strength between all indicators. Then, it was input into the FCM Analyst software, and the causal impact weight between the concept nodes could be automatically integrated by the software. The FCM dynamic model was established in the software. (3) Model application. The evolution analysis was carried out in FCM Analyst software, followed by prediction analysis.

### Exploratory Factor Analysis

Exploratory factor analysis (EFA) can identify the internal relationship between different variables, and classify several variables with close ties and high correlation into one category (Niu et al., 2019). EFA is used to address the problem of analyzing the interrelationships among a large number of variables (e.g., questionnaire responses) by defining a set of common underlying dimensions, known as factors. Based on principal component analysis and variance rotation, this study uses exploratory factor analysis to explore the internal relationship between the variables affecting the construction of intelligent transportation.

## Data Analysis

Five professionals were invited to conduct pilot studies to test and refine the initial variable table. These five experts are researchers, professors and construction engineers of intelligent transportation. They are qualified and experienced in both scientific and practical aspects. All professionals have participated in the construction of intelligent transportation in many cities and have more than 10 years of research experience in the field of transportation. Therefore, experts in the field can refine the initial variable table and judge the correlation between factors based on their work experience, which provides the possibility to validate the simulation results to some extent. The refined questionnaire was distributed to 518 professionals engaged in traffic construction. Comprehensive investigation began from 2022.01 to 2022.03. A total of 208 questionnaires were collected, of which 188 were valid (valid response rate was 36.29%). Forty-eight from academia and 140 from industry. They have relevant experience and knowledge in smart city construction and smart transportation construction. It means that the answers they provide are valid. Basic information of the respondents is shown in Table 2. The ratio of sample size to variables is about 9:1, which meets the conditions for exploratory factor analysis (the ratio of sample size to variables is higher than 5:1)(Kyriazos, 2018).

**TABLE 2:** Profiles of respondents.

Category	Classification	Numbers	Percentage (%)
Age	18–24	15	7.98
	25–30	38	20.21
	31–40	59	31.38
	>40	76	40.43
Years of experiences	<5	64	34.04
	5–10	83	44.15
	>10	41	21.81
Education level	Junior College and below	55	29.26
	Bachelor	55	29.26
	Master	63	33.50
	Ph. D and above	15	7.98
Position	General staff	60	31.91
	Project manager	51	27.13
	Department manager	40	21.28
	Senior manager	37	19.68

The test result also indicated that the sample was well suited for this method, with the Kaiser–Meyer–Olkin (KMO) index reaching 0.870 and with Bartlett’s test rejecting the null hypothesis (χ^2^ = 1688.73, *df* = 264, sig. = 0.000) as shown in Table 3. Cronbach’s α coefficient ranging from 0.904 to 0.908 showed that each extracted factor was internally consistent. By the latent root criterion, a six-component finding was carried out, with a total variance of 61.339% (>60%), which adequately relied on the Malhotra guideline (Zhang et al., 2019). Each variable only loaded heavily on one of the six factors, with a weight above 0.50. Item communalities were all higher than 0.4, showing that the variables were explained well by the underlying factors (Watkins, 2018).

**TABLE 3:** Results of exploratory factor analysis.

No.	Cronbach’s α	Component (Variable groupings)					
		1	2	3	4	5	6
V14	0.908	0.682					
V16	0.906	0.716					
V17	0.905	0.693					
V18	0.908	0.720					
V19	0.905	0.780					
V01	0.907		0.693				
V04	0.906		0.838				
V06	0.904		0.641				
V02	0.905			0.589			
V05	0.907			0.618			
V09	0.907			0.606			
V03	0.908				0.787		
V07	0.905				0.557		
V08	0.907				0.649		
V10	0.907					0.836	
V13	0.908					0.756	
V20	0.905					0.681	
V11	0.906						0.811
V12	0.907						0.751
V15	0.909						0.594
Variance (%)		13.02	12.31	10.76	10.07	8.31	7.37
Cumulative variance (%)		13.02	25.33	36.09	46.16	54.47	61.84
Kaiser-Meyer-Olkin measure of sampling adequacy							0.856
Bartlett’s test of sphericity			Approximate χ^2^				1688.73
			*df*				264
			Significant				0.000

For further discussion, it is necessary to rename the components. The practical significance of a component can be explained by the common characteristics of the integrated variables, or by the relatively high variables it carries. Considering the characteristics of smart transportation and following the internal relationship between variables, potential grouping can be better understood. These six components are named: (1) Policy support (PS), including V14, V16, V17, V18, V19; (2) Traffic sector control (TSC), including V01, V04, V06, (3) Technical support (TS), including V02, V05, V09; (4) Communication foundation (CF), including V03, V07, V08; (5) Residents’ recognition (RR), including V10, V13, V20; (6) Talent quality (TQ), including V11, V12, V15.

## Fuzzy Cognitive Map Model and Predictive Analysis

### Identification of Concept Nodes

Based on the previous analysis results, seven conceptual nodes, including policy support (PS), traffic sector control (TSC), technical support (TS), communication foundation (CF), residents’ recognition (RR), talent quality (TQ), and intelligent transportation construction (ITC), which were about to form the FCM model were identified.

### Causality Judgment and Weight Determination

Based on the experience and knowledge of the above five experts, experts were invited to use nine-level fuzzy semantics (see Table 4) for causal relationship judgment and scoring to describe the interaction between conceptual nodes. The scoring results were input into the FCM Analyst software, and the causal impact weights between concept nodes could be automatically integrated by the software (Zhang et al., 2017). For example, the influence of concept node *i* on *j* is scored, and the judgment results are respectively zero, negative medium, negative weak, negative weak, negative medium, negative weak, negative weak, negative weak, negative weak, negative weak and negative weak. After inputting the software in turn, the final causal influence weight is −0.275.

**TABLE 4:** The sources of the variables.

No.	Fuzzy semantics	Symbol	Value
1	Negative very strong	μ_*nvs*_	–1.0
2	Negative strong	μ_*ns*_	–0.75
3	Negative medium	μ_*nm*_	–0.50
4	Negative weak	μ_*nw*_	–0.25
5	Zero	μ_*z*_	0
6	Positive weak	μ_*pw*_	0.25
7	Positive medium	μ_*pm*_	0.50
8	Positive strong	μ_*ps*_	0.75
9	Positive very strong	μ_*pvs*_	1.0

Based on the FCM Analyst software, a summary of the weight scores of multiple experts was obtained. The calculation rules are set in the software and the user only needs to input the semantic judgments of the experts on the causal influences between the concept nodes. The calculation formula is as follows, where *W*_*ij*_ represents the comprehensive influence weight of concept node *i* to *j*; *M* represents the total number of participating experts; *b_k_* represents the credibility weight of the *k*th expert; *W_ij_^k^* represents the fuzzy influence weight of concept node *i* to *j* based on the judgment of the *k*th expert.


(1) Wij=∑k=1M(bk×Wijk)M

### Construction of FCM Model

**FIGURE 1:**
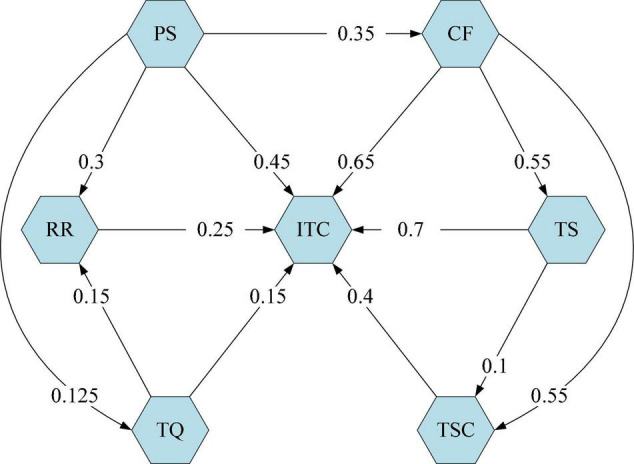
FCM Model of Intelligent Transportation Construction.

Based on the integration of the above five expert judgment results, the FCM model was finally formed by using the software (see Figure 1). To make the model an organic whole, a directed arc representing causality must be used to connect the cause and result nodes. At the same time, the weights of these directed arcs are set. Therefore, the model contains the causal relationship and weight between concept nodes. The values between seven concept nodes represent the causal impact weights, representing the extent to which concept node *i* affects *j*.

### Predictive Analysis

The meaning of predictive analysis is that when the existence of a concept node is found, it can predict future results. In this paper, it is used to analyze the changes and updates of intelligent transportation construction caused by the changes of influencing factors, in order to identify the direct causality. In FCM Analyst, the value of causality can be expressed by the five-point language scale of “very disadvantageous, unfavorable, neutral, advantageous and very advantageous”. In the initial phase, except for specific concept nodes, the values of all concept nodes are set to neutral 0.

**FIGURE 2:**
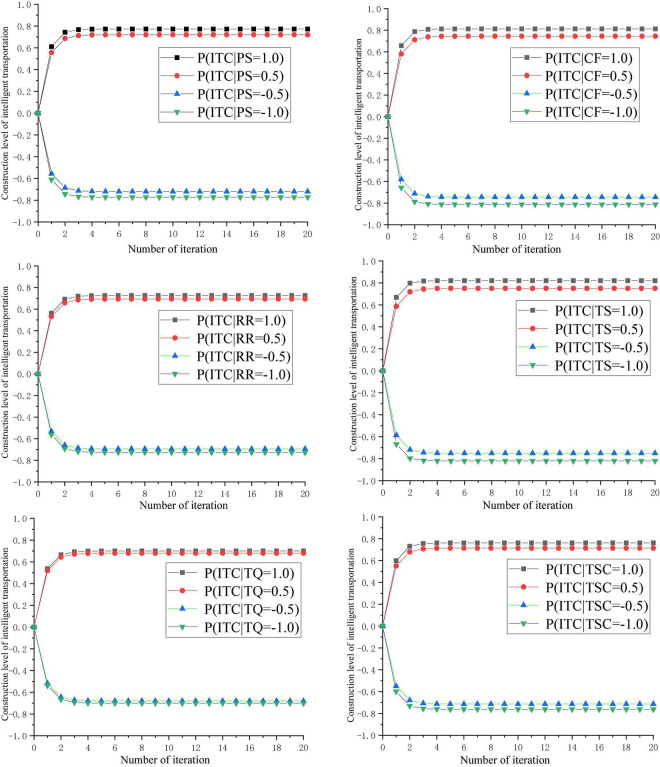
Impacts of variables on the ITC. Policy support (PS), traffic sector control (TSC), technical support (TS), communication foundation (CF), residents’ recognition (RR), talent quality (TQ), and intelligent transportation construction (ITC).

It was assumed that the *i*th concept node is at a very unfavorable level (−1.0), an unfavorable level (−0.5), a favorable level (+0.5), and a very favorable level (+1.0), respectively. The final result of intelligent transportation construction was obtained in these four situations. The target concept node (intelligent transportation construction) evolved continuously and stabilizes at a fixed value after many interactions. The impact of the change of concept nodes on intelligent transportation construction (ITC) was shown in Figure 2, and the fixed value of intelligent transportation construction after iteration in different situations was shown in Table 5. The selection of the maximum number of iterations in this study was based on the following. The dynamic model of FCM is essentially a multiplication operation between the vector composed of the initial state value of the index (concept node) and the matrix of the causal correlation strength between the index. Then a threshold function (the threshold function included in FCM Analyst software) was transformed to get the state value of the concept node at the next time. If the state value of the concept node does not reach the convergence condition at the next time, the iteration continues until the convergence is reached.

**TABLE 5:** Fixed values of ITC after a set of iterations in different scenarios in the predictive analysis.

Scenarios	*P*(ITC|*i* = + 1.0)	*P*(ITC|*i* = + 0.5)	*P*(ITC|*i* = −0.5)	*P*(ITC|*i* = −1.0)
PS	0.7725	0.7201	−0.7201	−0.7725
CF	0.8118	0.7445	−0.7445	−0.8118
RR	0.7264	0.6940	−0.6940	−0.7264
TS	0.8206	0.7503	−0.7503	−0.8206
TQ	0.7007	0.6803	−0.6803	−0.7007
TSC	0.7616	0.7138	−0.7138	−0.7616

Taking the concept node PS as an example, when the value of PS is 1.0 (or 0.5), the target concept node ITC is stable at a fixed value of 0.7725 (or 0.7201) after several iterations, which indicates that there is a significant positive correlation between PS and ITC. Similarly, the concept node CF, RR, TS and TQ are significantly positively correlated with the target concept node TSC. In terms of correlation intensity, the ranking of the six dimensions is TS, CF, PS, TSC, RR, and TQ. Among them, technical support (TS), communication foundation (CF) and policy support (PS) have the strongest positive correlation with intelligent transportation construction (ITC), which are the core influencing factors of ITC. Therefore, full attention should be paid to the development of technology related to intelligent transportation, the establishment of communication foundation and the improvement of supporting policies in the construction and management of intelligent transportation.

## Discussion

Urban transportation system consists of several subsystems. The level of development of a city’s transportation is closely related to its historical reasons, natural conditions, policy support, science and technology, and citizens’ demands. Based on the system analysis of intelligent transportation construction, countermeasures and suggestions are made for the development of urban intelligent transportation construction.

(1) Technologies related to the development of intelligent transportation construction should be vigorously developed to accelerate the landing of technical achievements and promote the development of urban intelligent transportation industry. Breakthroughs in key technologies are the guarantee of the development of intelligent transportation construction. Intelligent transportation will be cloud computing, Internet, big data and other advanced technologies and cutting-edge information technology into one. This realizes the use of vehicle networking in transportation and is changing the way people travel. This provides new ideas to promote the development of urban intelligent transportation construction.
(2) Whether the city’s transportation supply can meet the public’s demand for transportation is an important indicator of the public’s satisfaction with travel. It is also the key to check whether it can be recognized by the public and whether satisfactory smart transportation can be built. The application of wisdom facilities and infrastructure to transportation can improve the efficiency of transportation operation. Therefore, the communication foundation will affect the transportation supply capacity of Qingdao city under the policy promotion. The means of communication and information technology can provide better services to citizens and create a good living environment.
(3) The current intelligent transportation construction in many cities is in the initial stage, and a perfect top-level design has not yet been formed. The development of urban intelligent transportation construction must rely on the government’s assistance and support. National policies largely affect the development of urban intelligent transportation construction. Therefore, improving the support policy and increasing support are beneficial to the development of urban intelligent transportation construction.

## Conclusion

With the booming development of global smart cities, the construction and development of urban intelligent transportation is facing opportunities and challenges. This study explored the key influencing factors of urban intelligent transportation construction and proposed a root cause analysis method based on fuzzy cognitive map (FCM) to model intelligent transportation construction. To explore the potential group among all the 20 variables, an exploratory factor analysis was conducted. The results showed that there are six impacted sources on urban smart transportation construction, including policy support (PS), traffic sector control (TSC), technical support (TS), communication foundation (CF), residents’ recognition (RR), talent quality (TQ). In the FCM model, all six concept nodes have a significant positive correlation with the target concept node TSC. The rank of the six dimensions based on correlation strength is TS, CF, PS, TSC, RR, and TQ. The most probable root indexes are technical support (TS), communication foundation (CF) and policy support (PS), respectively. Therefore, early intelligent transportation-related technologies should be innovated and integrated, communication infrastructure should be improved, and appropriate policy support should be formulated.

In summary, this study revealed the impacted sources on urban intelligent transportation construction, which have important management significancy for the practice. The results of the study showed that the factor framework has good applicability and operability.

The concern of this study is that the majority of respondents in the practitioner group are mainly engaged in smart city and smart transportation construction in developing countries. This is generally acceptable as the mutual learning on the macro level is existed, although the level of smart transportation construction varies from country to country internationally. It should be noted that this study only focuses on the key variables that contribute to the construction of smart transportation in cities and their ultimate impact on the construction of smart transportation. Further research will be conducted to reveal the interrelationships among these variables and the dynamic interactions among them. This will help intelligent transportation builders understand how to pursue construction speed and quality in the best way.

## Data Availability Statement

The original contributions presented in this study are included in the article/supplementary material, further inquiries can be directed to the corresponding authors.

## Author Contributions

LZ: conceptualization, methodology, software, formal analysis, writing original draft, validation, and investigation. QW: supervision and modification. B-GH: supervision. All authors contributed to the article and approved the submitted version.

## Conflict of Interest

The authors declare that the research was conducted in the absence of any commercial or financial relationships that could be construed as a potential conflict of interest.

## Publisher’s Note

All claims expressed in this article are solely those of the authors and do not necessarily represent those of their affiliated organizations, or those of the publisher, the editors and the reviewers. Any product that may be evaluated in this article, or claim that may be made by its manufacturer, is not guaranteed or endorsed by the publisher.
